# Differences in childhood stress between Neanderthals and early modern humans as reflected by dental enamel growth disruptions

**DOI:** 10.1038/s41598-024-61321-x

**Published:** 2024-05-23

**Authors:** Laura Sophia Limmer, Matteo Santon, Kate McGrath, Katerina Harvati, Sireen El Zaatari

**Affiliations:** 1 Paleoanthropology, Senckenberg Centre for Human Evolution and Palaeoenvironment, Institute of Archaeological Sciences, University of Tübingen, Tübingen, Germany; 2 DFG Center of Advanced Studies ‘Words, Bones, Genes, Tools: Tracking Linguistic, Cultural and Biological Trajectories of the Human Past’, Tübingen, Germany; 3 Ecology of Vision Group, University of Bristol, Bristol, UK; 4 Department of Anthropology, Center for the Advanced Study of Human Paleobiology, The George Washington University, Washington, DC, USA; 5 CENIEH, Burgos, Spain

**Keywords:** Biological anthropology, Archaeology

## Abstract

Neanderthals’ lives were historically portrayed as highly stressful, shaped by constant pressures to survive in harsh ecological conditions, thus potentially contributing to their extinction. Recent work has challenged this interpretation, leaving the issue of stress among Paleolithic populations highly contested and warranting in-depth examination. Here, we analyze the frequency of dental enamel hypoplasia, a growth disruption indicator of early life stress, in the largest sample of Neanderthal and Upper Paleolithic dentitions investigated to date for these features. To track potential species-specific patterns in the ontogenetic distribution of childhood stress, we present the first comprehensive Bayesian modelling of the likelihood of occurrence of individual and matched enamel growth disruptions throughout ontogeny. Our findings support similar overall stress levels in both groups but reveal species-specific patterns in its ontogenetic distribution. While Neanderthal children faced increasing likelihoods of growth disruptions starting with the weaning process and culminating in intensity post-weaning, growth disruptions in Upper Paleolithic children were found to be limited around the period of weaning and substantially dropping after its expected completion. These results might, at least in part, reflect differences in childcare or other behavioral strategies between the two taxa, including those that were advantageous for modern humans’ long-term survival.

## Introduction

Neanderthals have been traditionally portrayed as having led exceptionally stressful lives including the pressure to survive in the harsh and widely fluctuating ecological conditions of Pleistocene Eurasia, which was thought to have contributed to their extinction^1^. Even though Upper Paleolithic modern humans (UPMH) also faced similar environmental conditions, particularly leading up to and during the Last Glacial Maximum^2^, they are commonly believed to have been better able to mitigate such pressures through their behavioral repertoire. This included strategies such as greater flexibility and efficiency in resource exploitation and more complex social organization and networks. Their behavioral repertoire was thought to have provided UPMH with a competitive advantage over Neanderthals, allowing them to persist while Neanderthals perished^3–7^. Some recent studies, however, are casting doubt on this view, arguing instead that Neanderthals and UPMH led similarly stressful lives^8–10^.

Here, we further explore the topic of stress in the Paleolithic. We track dental enamel hypoplasia, i.e., localized areas of reduced enamel thickness resulting from periods of growth disruptions during crown formation^11–13^. Experimental and clinical research on recent humans, primates, and on other mammals, has extensively shown that physiologically demanding periods, such as times of illness, infections, malnutrition, nutritional deficiencies, or trauma, can result in the manifestation of hypoplastic defects in teeth^11,14,15^. Additionally, since enamel is deposited with known regularity and does not remodel in later life, the vertical position of such hypoplastic defects within tooth crowns can also be indicative of their timing of occurrence and possibly duration in dental developmental terms^11,16,17^. Examinations of dental enamel hypoplasia can thus be used to provide insights into stressful periods during childhood development. Given the vast etiology of dental enamel hypoplasia and the variability of potential stressors^11,13,18–21^, we follow here Temple and Goodman^22^, in using the term “developmental or childhood stress” to describe a physiological disruption sufficient to affect hard tissue formation.

The potential of using enamel growth disruptions, particularly in their linear form (LEH), to elucidate stress profiles of past populations has prompted many studies to track enamel hypoplasia occurrences in Paleolithic hominins^10,23–31^. However, these have provided contradictory results when it comes to reconstructions and comparisons of stress levels in Neanderthals and UPMH. While some concluded that Neanderthal children were subjected to severe levels of physiological stress^28,30^, i.e., higher than those observed in UP or recent modern humans^23^, others argued that the levels documented for Neanderthals were comparable to those seen in UPMH^9^ and also fell within expected ranges of modern foraging populations^25,27^. The lack of agreement in the interpretations of previous studies stems mostly from two factors: the focus on different samples in different studies, and/or their use of different methodologies. The samples in previous studies have often been limited in the number of individuals or sites represented. Moreover, inconsistencies in the methodology and objectives of these studies, e.g., differences in the kinds of defect and tooth types included, are limiting the potential for direct comparisons. As a result, there is a lack of a comprehensive understanding of enamel hypoplasia occurrences in Paleolithic hominins and thus their implications for childhood stress. Additionally, only a few studies have focused on hypoplastic defects in the context of their ontogenetic distribution^26,28,29,32^. Thus, our understanding of the relationship between stress marker occurrence and life history and development remains limited.

**Figure 1:**
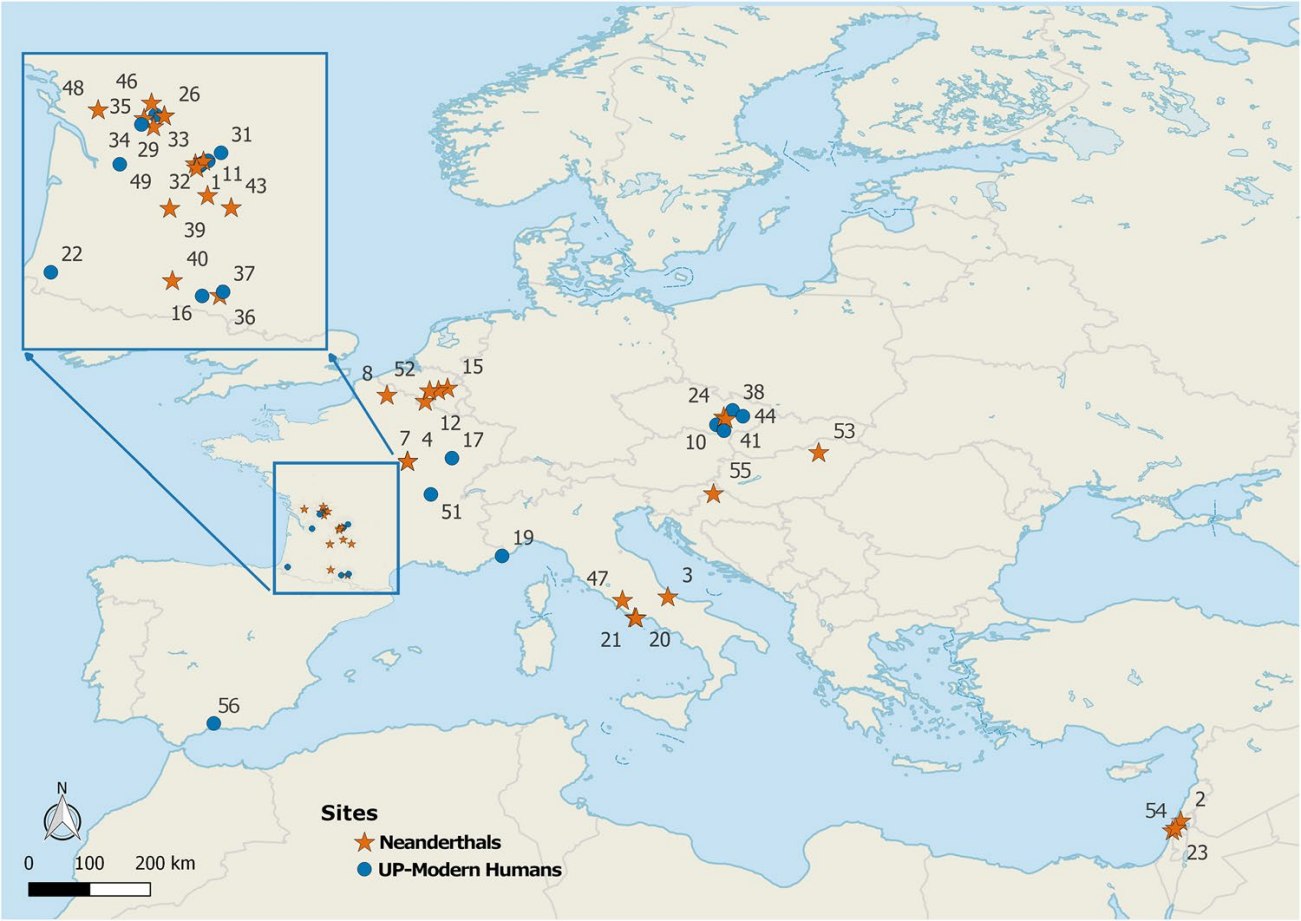
Sites included in this study. The Map was created in QGIS v.3.18 using Natural Earth vector map data. (1) Abri Pataud, (2) Amud, (3) Archi, (4) Arcy-Sur-Cure Grotte Bison, (5) Arcy-Sur-Cure Grotte De L'hyène, (6) Arcy-Sur-Cure Grotte Des Fées, (7) Arcy-Sur-Cure Grotte Du Renne, (8) Biache-St-Vaast, (9) Blanchard (Castelmerle), (10) Brno, (11) Combe Grenal, (12) Couvin, (13) Cro-Magnon, (14) Dolní Věstonice, (15) Engis, (16) Estelas, (17) Farincourt, (18) Fontéchevade, (19) Grimaldi-Barma Grande, (20) Grotta Breuil, (21) Guattari, (22) Isturitz, (23) Kebara, (24) Kulna, (25) La Chaise-Abri Bourgois-Delaunay, (26) La Chaise-Suard, (27) La Ferrassie, (28) La Madeleine, (29) La Quina, (30) Labatut (Castelmerle), (31) Lachaud, (32) Laugerie-Basse, (33) Le Moustier, (34) Le Petit Puymoyen, (35) Les Rois, (36) Malarnaud, (37) Masd'Azil, (38) Mladeč, (39) Monsempron, (40) Montmaurin, (41) Ochoz, (42) Pavlov, (43) Pech De L'Aze, (44) Predmostí, (45) Roc De Marsal, (46) Rochelot, (47) Saccopastore, (48) Saint Césaire, (49) Saint-Germain-La-Rivière, (50) Scladina, (51) Solutré, (52) Spy, (53) Subalyuk, (54) Tabun, (55) Vindija, (56) Zafarraya.

Here, we build upon previous work, overcoming these limitations. We assess the frequency and ontogenetic distribution of dental growth disruptions in the largest sample of Middle and Upper Paleolithic remains studied to date for this purpose, i.e., a total of 867 teeth representing 176 individuals from a total of 56 sites (Fig. 1, Supplementary Data File).

As growth disruptions can be expressed in a different way in different tooth types^12,16,21,33^, we follow the approach proposed by Towle and Irish^20^. We thus employ a comprehensive approach, assessing all hypoplastic defects (i.e., LEH/furrow form, pits/lines of pitting, localized hypoplasia of the primary canine^11,13^) and, for the linear form, also tracking their developmental timing across all tooth types. We aim to investigate: (1) whether Neanderthals and UPMH experienced different levels of enamel growth disruptions during development and (2) whether the ontogenetic distribution of enamel hypoplasia as a marker for potential physiological stress differed between the two taxa.

## Results

To fulfill these aims, we implemented two generalized linear mixed models. We first evaluated the overall likelihood of enamel hypoplasia occurrence for Neanderthals and UPMH in Model 1. Within hominin groups, we assessed the likelihood for defect manifestation on any single tooth, regardless of its type. Then, we used this same Model 1 to assess the ontogenetic distribution of the likelihood of enamel defect manifestation in each tooth type of each taxon by referencing their known developmental schedules.

We further explored the ontogenetic pattern of defect occurrence in Model 2, but here, instead of single teeth, we focused on multiple teeth of single individuals, where we tracked the likelihood of occurrence of systemic stress, i.e., single, or closely timed stress events, as manifested by hypoplastic defects on simultaneously forming regions of different tooth types of single individuals.

### Assessment of the likelihood of hypoplastic defect manifestation in single teeth within each taxon—Model 1

**Figure 2:**
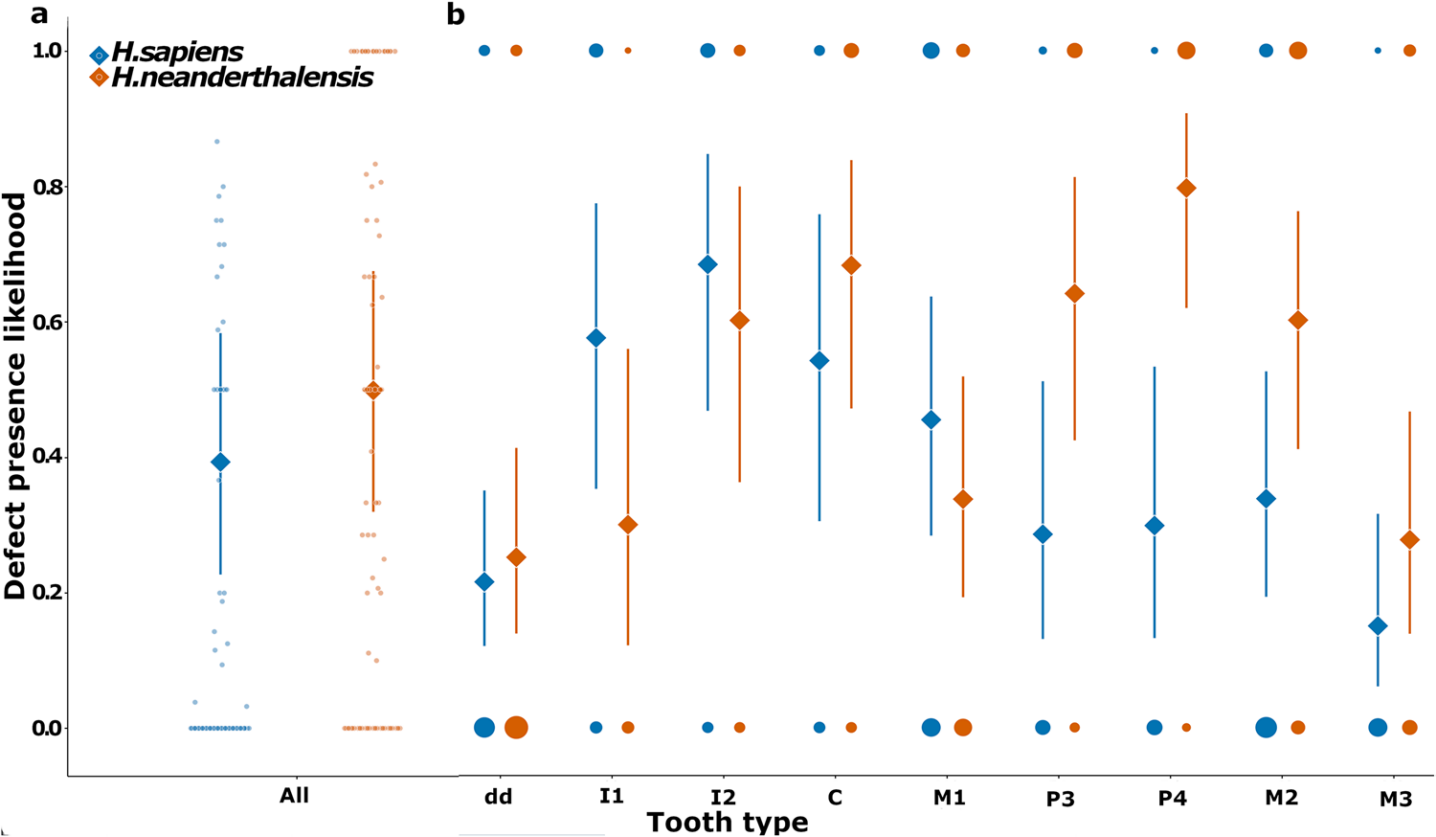
likelihood of Neanderthal and UPMH teeth being affected by enamel hypoplasia (a) when considering all tooth types in combination, and **(b)** when considering different tooth types separately (deciduous teeth: dd with different tooth types combined due to small sample sizes; permanent teeth: I = incisors, C = canines, P = premolars, M = molars). Colored points in (**a**) represent the likelihood of defect presence averaged for tooth types. Colored points in (**b**) represent the density of observations for each combination of hominin and tooth type. Diamonds in (**a,b**) represent model predicted medians with error bars showing their 95% credible intervals.

Our results for Model 1 reveal that, irrespective of tooth type or defect form, Neanderthal and UPMH teeth are comparably likely to be affected by hypoplastic defects (Fig. 2a, Supplementary Tables S1 and S2).

When the likelihood of hypoplastic defect manifestation was calculated by tooth type, the results show that, within Neanderthals, the deciduous teeth were overall less likely to show defects than all permanent tooth types, except for central incisors, first molars, and third molars, from which no considerable differences were detected (Fig. 2b, Supplementary Table S3). Within the Neanderthal permanent dentition, the central incisors, and first and third molars were less likely to exhibit defects compared to the canines, premolars, and second molars. Additionally, the central incisors were also less likely to exhibit defects compared to the lateral incisors, while the latter were less likely to exhibit defects in comparison to fourth premolars (Fig. 2b, Supplementary Table S3).

In UPMH, deciduous teeth were less likely to show hypoplastic defects compared to the permanent central and lateral incisors, canines, and first molars (Fig. 2b, Supplementary Table S3). Within the UPMH permanent dentition, lateral incisors were more likely to exhibit enamel hypoplasia compared to the premolars and second and third molars. The third molars were considerably less likely to show defects than all permanent tooth types except for premolars and second molars (Fig. 2b, Supplementary Table S3).

Finally, when we compared the likelihood of defect occurrence by tooth type between the two hominin groups, only premolars and second molars showed a higher likelihood of defect presence in Neanderthal teeth than in UPMH (Fig. 2b, Supplementary Table S2).

### Assessment of the likelihood of systemic stress marker occurrence throughout ontogeny—Model 2

In Model 2, we assessed and compared the likelihoods of Neanderthal and UPMH individuals experiencing systemic stress as manifested in hypoplastic defects at each of our predefined 11 dental developmental stages (Table 1, Supplementary Table S4).

**Table 1:** Dental development stages for model 2.

Developmental stage	Crown formation status in thirds	Equivalent biological age ranges in modern Northern Europeans	Equivalent biological ages and age ranges published for Neanderthals	Sample NEA (total 49 matched stress episodes in 35 individuals)	Sample UPMH (total 31 matched stress episodes in 37 individuals)
1	Deciduous dentition^a^			1	0
2	1/3: I^1^, I_1_, I_2_, M^1^, M_1_	1–1.8 years	0.65–1.53 years	0	1
3	2/3: I^1^, I_1,_ I_2_, M^1^, M_1_ 1/3: I^2^, C^x^, C_x_	1.5–2.9 years	0.81–2.67	5	12
4	3/3: I_1_, I_2_, M^1^, M_1_ 2/3: I^2^, C^x^, C_x_ 1/3: P^3^, P_3_	2.1–3.7 years	1.47–2.75	7	9
5	3/3: I^1^, I^2^ 2/3: P^3^, P_3_ 1/3: P^4^, P_4_	2.9–4.6 years	2.67–3.88	9	1
6	3/3: C^x^, C_x_, P^3^, P_3_ 2/3: P^4^, P_4_ 1/3: M^2^, M_2_	3.4–5.6 years	2.37–3.87 Engis 2 (3.0 years)	15	5
7	3/3: P^4^, P_4_ 2/3: M^2^, M_2_	4.4–5.5 years	Gibraltar 2 (4.6 years)	9	1
8	3/3: M^2^, M_2_	5.1–5.9 years	Krapina Maxilla B (5.9 years)	0	2
9	Crown developmental hiatus			0	0
10	1/3: M^3^, M_3_	9.4–9.7 years		0	0
11	2/3–3/3: M^3^, M_3_	9.6–10.9 years	Scladina I-4A (8 years)	3	0

Definition of stages was based on the concurrent formation of tooth crown thirds in modern Northern Europeans^54,63^. Also provided are corresponding biological ages in the same Northern European sample as well as in Neanderthals as available from the anterior dentition (from I^1^, I^2^, C^x^, C_x_ and combining estimates for both 7 and 8 day periodicity periods)^26^. Neanderthal juvenile individuals with direct dental histological age-at-death estimations are included within their corresponding dental developmental stage as an additional reference^49^.

Permanent teeth: *I* incisors, *C* canines, *P* premolars, *M* molars.

^a^All deciduous teeth formation stages are lumped in one stage due to small sample sizes.

**Figure 3:**
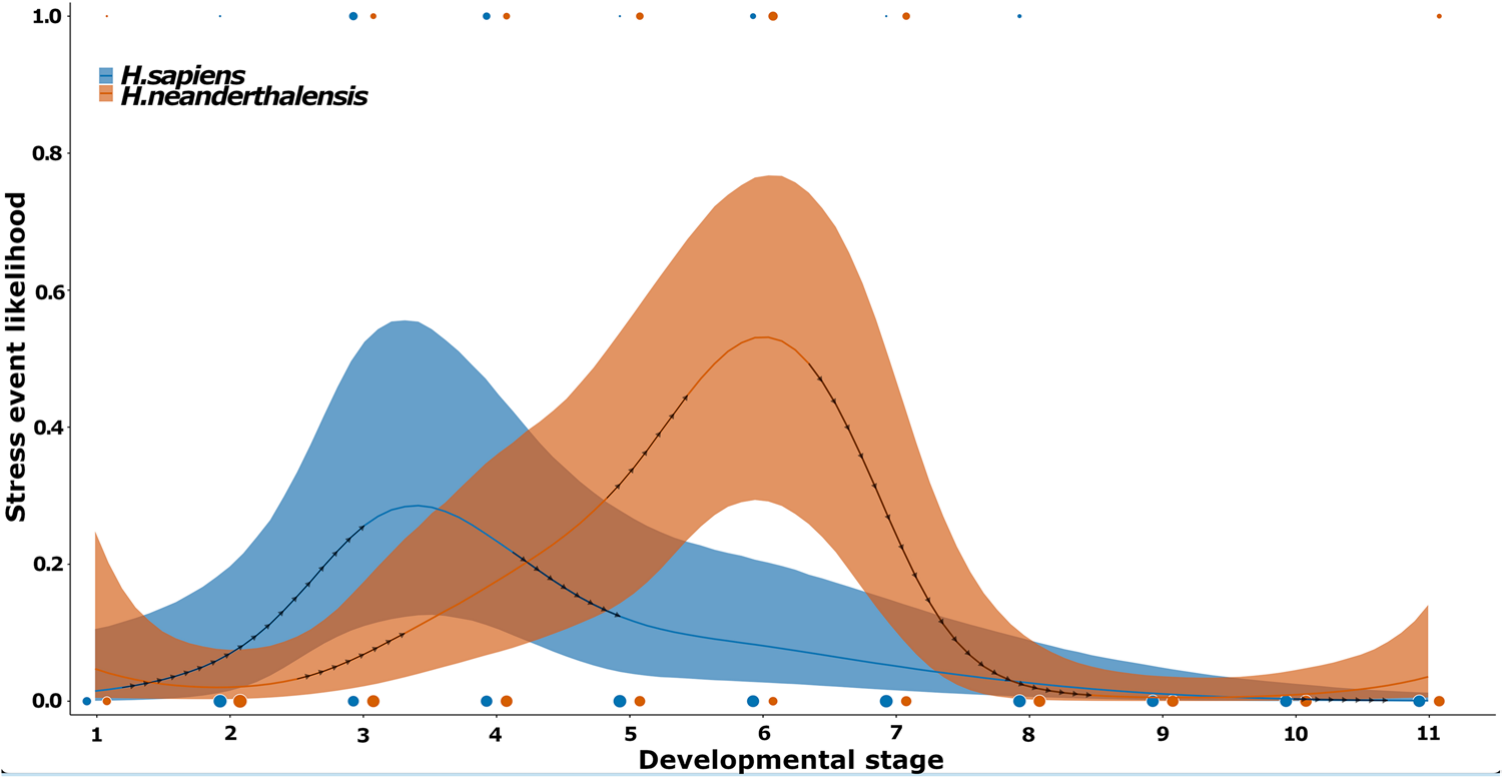
Likelihoods of Neanderthal and UPMH individuals experiencing systemic stress occurrences throughout predefined developmental stages. Lines represent model predicted medians of stress event occurrences likelihoods across 11 developmental stages and shaded areas represent their 95% credible intervals. Black arrows represent periods of marked increase or decrease in the likelihood of stress event occurrence. Colored points represent the density of observations for each combination of hominin and developmental stage. A description of the developmental stages, distribution of stress events and the equivalent biological age ranges in modern humans and Neanderthals are given in Table 1.

In the UPMH sample, the likelihood of occurrence of systemic stress episodes shows a marked increase starting between Stages 1 and 2 (as indicated by the black arrows in Fig. 3), which, after a peak shortly after Stage 3, is followed by a sharp and then gradual but steady decrease. In contrast, the Neanderthal curve shows the beginning of an increasing trend after Stage 2 that peaks at Stage 6, after which it drops sharply to culminate in a period with no traceable systemic growth disruptions after Stage 8. At the stages where the likelihood of hypoplasia occurrence peaks in each of the hominin groups, the recorded likelihood at the same ontogenetic stage for the other group remains relatively lower. At dental developmental Stage 6, the likelihood of hypoplasia occurrence in Neanderthals is considerably higher than that recorded in UPMH (Supplementary Table S5).

## Discussion

The first question this study set out to answer was whether Neanderthal and Upper Paleolithic children faced different levels of developmental stress as gleaned through the analysis of enamel growth disruptions. Previous analyses provided contradictory results, mainly owing to sample size limitations and sample and methodological diversity across studies. Our results are derived from the most comprehensive sample and assessment used to date. They reveal that the probability for any single tooth to manifest a hypoplastic defect (of any form) is similar in Neanderthals and UPMH. These results support previous work arguing for the lack of substantial differences in overall childhood stress levels between the two hominin groups^8–10^. Our findings therefore counter arguments that Neanderthal lives were generally much more stressful compared to those of UPMH.

Second, we addressed the question of whether Neanderthals and UPMH showed different patterns in the ontogenetic distribution of physiological stress as indicated by enamel hypoplasia. For this, we assessed the timing of defects occurrences by employing two approaches: for the first we referenced enamel hypoplasia occurrences on single teeth using the sequential development of crowns of different tooth types and for the second we matched systemic stress markers occurrence for individuals to one of 11 developmental stages. These stages were defined based on the combination of crown formation status of concurrently developing tooth types.

When comparing tooth types, we found that deciduous teeth stand out in both hominin groups as being generally less likely to manifest enamel defects relative to the permanent dentition. This is consistent with previous reports of low defect prevalence in the primary dentition, not only in Paleolithic but also in recent modern human groups^11,28,31,34,35^. Yet, overall, compared to permanent dentition, enamel defects in deciduous teeth in human groups are not-well documented^21,31^. It is possible that different enamel properties and growth patterns of deciduous and permanent teeth could make the former relatively less prone to enamel defects; deciduous teeth generally have faster-forming enamel, resulting in shorter developmental windows and fewer perikymata manifested on the enamel surface^11,13,18,36^. However, it is also possible that such low prevalence might reflect a sheltered fetal environment during the formation of deciduous tooth crowns^11^.

Our results also reveal that Neanderthals and Upper Paleolithic modern humans differ in their pattern of likelihood of defect occurrence among permanent tooth types. While in UPMH the central and lateral incisors are the most likely tooth types to show enamel defects, in Neanderthals, it is canines and premolars instead. In fact, the premolars (and second molars) are considerably more likely to show defects in Neanderthals compared to UPMH. As the crown formation times of permanent incisors do not generally overlap with those of premolars and second molars in either hominin group^21,37,38^ (Table 1), our results allow us to distinguish a developmentally earlier peak in stress-related enamel defect prevalence in UPMH versus a developmentally later one in Neanderthals.

However, single teeth are prone to individual and population specific variation in their enamel growth, microstructure, and crown geometry, all of which might affect their level of susceptibility to disruptions^11,13,17,39^. We thus cross-checked our results using a second approach in which we concentrated on linear defects matched across multiple tooth types with overlapping crown formation times in single individuals to track systemically stressful periods throughout ontogeny. Linear enamel hypoplasia in particular has been associated with non-specific stress^11–13^. Using this second approach, we also found distinctive patterns of ontogenetic defect distribution in Neanderthals and UPMH **(**Fig. 3**)**.

We observed that in the UPMH sample, the likelihood of systemic hypoplastic manifestations starts to increase between our dental developmental stages 1–2. This likelihood continues to sharply increase, to peak at a stage of dental development (our Stage 3–4) coinciding with the time of formation of the last third of the permanent incisors and first molar crowns (Fig. 3; Table 1). Therefore, the initiation of the increase in likelihood of stressful periods and its peak, corresponds to the average biological ages at which two life history milestones related to the process of weaning are documented among non-industrial populations^40^: the average age of first introduction of solid foods around 6 months; and the average age of completion of the weaning process around 2.5 years (Table 1). A number of studies have demonstrated a link between the stressful process of weaning in the first years of life and the formation of linear enamel hypoplasia particularly on the anterior teeth in nonhuman primates^41–43^. Such a relationship has also been hypothesized for hominins^11,16,28,29,32,34,44–46^. During the weaning process, the increasing energetic demands of a growing infant must be met by supplementing breastmilk with foods that provide the needed nutrition; otherwise, insufficient nourishment can lead to malnutrition, chronic digestive problems, and an increased risk of disease, all potentially causing high metabolic stress, growth disruptions, and thus peaks in enamel hypoplasia occurrences^28,34,47^.

In UPMH children, the period coinciding with the process of weaning (from its initiation to its completion) apears to have been the most stressful time, after which we document a gradual continuous decrease in defect occurrence likelihood. In Neanderthals, the pattern is notably different. First, compared to UPMH, the initiation of the trend of increased stress seems to be delayed by around a stage and a half of dental development (Fig. 3). Similarly, at dental developmental Stages 3–4, when the hypoplastic manifestation of stress peaks in UPMH, such manifestations are still considerably lower in Neanderthals. For the latter, the likelihood of hypoplastic manifestations of stressful periods increases to the peak level seen in UPMH midway between dental developmental Stages 4–5 (Fig. 3). Additionally, beyond this point in ontogeny, Neanderthals continue to increasingly be at risk of experiencing further systemic stress events, with likelihoods ultimately surpassing the highest levels we observe for the UPMH, and peaking at a later stage of dental development, i.e., at Stage 6 (after the completion of the incisor and first molar crowns, when the second molar crowns are starting to form). At this point, the likelihood of stress manifestation occurrences in UPMH has already significantly dropped.

This ontogenetically delayed initiation and peak in stress events in Neanderthals might initially be interpreted as reflecting delayed initiation and completion of the weaning process in comparison to UPMH. However, dental development could potentially be accelerated in Neanderthals compared to modern humans^48–50^. In such a case, and following dental crown developmental charts established for Neanderthals^26^, the initiation of a trend of increasing likelihood of manifestation of stressful episodes can be aged at between 6 and 9 months **(**Table 1). This also coincides with the age at introduction of solid foods evidenced in several Neanderthal individuals^28,45,51–53^. Similarly, also following the age estimates from Neanderthal dental crown developmental charts^26^, as well as more accurate age determinations for relevant specimens (i.e., Engis) based on dental histology^49^, the peak stress we observe in Neanderthals at our Stage 6 corresponds to a chronological age of around 3 years, thus younger than the corresponding estimate of 4.5 years of age for Northern European modern humans at this dental developmental stage^54^. Even though this biological age of 3 years is broadly within the average age range of 2.5 years ± 10 months for cessation of breastfeeding reported in non-industrial human populations^40,55,56^, available determinations of ages at cessation of breastfeeding for Neanderthal specimens show that the completion of weaning occurred at around 1.2 and 2.5 years^51,53^. With these estimations, we would then expect a stress peak related to the weaning process to manifest in Neanderthals at our dental developmental stages 4–5, which is not the case (Table 1). Thus, unless the weaning ages reported for these Neanderthal individuals significantly divert from the population average, the later peak we observe in this group (at our Stage 6) can be interpreted as a post-weaning signal of systemic physiological stress.

The early post-weaning phase would potentially continue to be a stressful stage as a child’s growing energetic demands, the developing immune system, and the increased independence, pose an increased risk of malnutrition and disease^19,47,57,58^ which would translate into an increased likelihood of hypoplastic defect manifestation. The observation of a reduction of physiological stress post-weaning in UPMH might thus indicate the presence of social and behavioral strategies and/or life history traits that would ensure sufficient high energy nutrition for newly weaned children^19,47^. Some such strategies that play a role in reducing early childhood physiological stress, like prolonged post-weaning dependency, optimized resource exploitation^4,5,7,52,59^, support in provisioning^47,55,58,60^ are believed to have been in place in the Upper Paleolithic, and could have contributed in turn to long-term advantages for the population^47,55,60^.

In summary, even though we detect a similar overall signal in hypoplasia occurrence likelihood on a taxonomic level between Neanderthals and UPMH, our findings suggest differences in the likelihood of occurrence of these defects throughout ontogeny between these two Paleolithic hominin groups. We interpret the latter as reflecting a better ability of UPMH in mitigating stress in newly weaned children, in contrast to Neanderthals where the period shortly after the presumed completion of the weaning process coincides with the most stressful childhood phase. Our results could thus be taken as implications for the practice of advantageous survival strategies by UPMH.

### Methods

This study is based on the examination of high-resolution epoxy replicas of 1048 Paleolithic deciduous and permanent dental remains from the Paleoanthropological collection, University of Tuebingen (Germany). Out of these, a total of 867 teeth of which 423 belonged to Neanderthals (n = 74 individuals) and 444 to UPMH (n = 102 individuals) were judged to be sufficiently preserved (with at least 50% of their crown height preserved and remnant enamel surfaces in good condition^20^) to be included in the analyses. These originated from a total of 56 western Eurasia sites (Fig. 1; Supplementary Data File).

Following established methodologies^11,12,26^, lingual and buccal/labial crown enamel surfaces of all selected teeth were inspected by one of the authors (L.S.L.) for the presence of hypoplastic defects under oblique light conditions, first with the naked eye and then with a 20× magnification lamp. Every single hypoplasia incidence identified, (i.e., LEH/furrow form, pits/lines of pitting, localized hypoplasia of the primary canine)^11,13^, was recorded; with the tooth type it affected also noted. In the cases when linear defects were identified on multiple teeth of single individuals, their horizontal locations within crown vertical thirds were additionally documented. These locations were then used to estimate ontogenetic timing of defect formation in terms of dental developmental stages. Dental developmental stages, rather than their corresponding biological ages in recent human populations, were used in this study as recent human standards might not be applicable to fossil hominins. In the particular case of Neanderthals, there are arguments that they might have accelerated dental growth rates compared to modern humans^48–50^. However, even if this is indeed the case, Neanderthals still seem to have followed a sequence of crown formation comparable to that of modern humans^26,49,52,61,62^. Thus, the use of dental developmental stages would mitigate any potential effects of differences in dental growth rates between Neanderthals and modern humans and would allow for comparisons of likelihoods of hypoplasia occurrence at a comparable developmental stage.

For this study, we defined a total of 11 dental developmental stages based on concurrency in development of crown third regions across different tooth types. For the permanent dentition, we identified which crown thirds were overlapping in their development using the age estimates within Reid and Dean^54^ and Holt et al.^63^ for dentitions of recent northern Europeans as a reference. We then defined dental developmental stages such that any one stage would mark the completion of formation of at least a third of the crown of one tooth type (Table 1). This grouping resulted in seven separate dental developmental stages of the permanent dentition (our Stages 2–8) starting from the beginning of formation of the crowns of the incisors to the completion of the formation of the crowns of the second molars. The gap in dental crown development between the time of completion of the second molar crowns and the initiation of formation of the third molar crowns was accounted for by allocating a separate stage (Stage 9) to it. This was also necessary for the statistical modelling. Our Stage 10 coincided with the time of formation of the first third of the third molar crowns; whereas the formation of the remaining two thirds were allocated to a single stage (Stage 11) due to the limited number of individuals with hypoplasia detected on these latter two thirds of the third molars in our sample. Similarly, due to the small sample size, crown development of all deciduous dentition was represented by a single stage (Stage 1).

For reference, we included in Table 1 chronological age range estimates for our stages for the modern northern European sample as derived from Reid and Dean^54^ and Holt et al.^63^ as well as for Neanderthals as available for some tooth types^26^. Additionally, available direct age-at-death determinations of juvenile Neanderthals using dental histological analyses^49^ were added in Table 1 to provide general comparisons. Even though chronological age ranges diverged, the documented sequential development of crown thirds in Neanderthals appear to match that of the modern northern European sample. Thus, for all fossil individuals, linear enamel defects reported on multiple (2 or more) teeth of single individuals and assigned to the same developmental stage were considered as broadly contemporaneous and taken to represent a stressful period, whether a single event or a series of closely timed events.

### Statistical analysis

Data analysis focused on assessing differences in the rate and timing of defect manifestations in the hominin groups using generalized linear mixed models (GLMMs). Models were implemented in R (version 4.2.1) with the brms package, which fits Bayesian models using Stan^64–66^. Two GLMMs were generated based on a Bernoulli distribution with logit-link to compare, between Neanderthals and UPMH, the likelihood of: (1) a tooth manifesting at least one hypoplastic defect of any form for each tooth type and for an average of all tooth types combined, and (2) an individual experiencing a stressful period at each of the predefined 11 developmental stages. Both models included *taxon* (NEA, UPMH) as the main factor predictor and *individual* and *site ID* as random components to account for the repeated measurements of single individuals and/or sites^67^.

Model 1 further included the factor predictor *tooth type* and the interaction between *tooth type* and the main factor predictor *taxon*. Permanent teeth were divided by tooth type (I1, I2, C, P3, P4, M1, M2, M3) albeit without regard to side or jaw to increase sample sizes, while deciduous teeth were combined into a single category due to low sample sizes when split by type. This approach allowed for the inclusion of all teeth within our sample (N = 867 teeth of which 423 belong to 74 Neanderthals and 444 to 102 UPMH), including those that were found in isolation.

Model 2 included, as a covariate, a smooth interaction term based on the standardized continuous predictor *developmental stage* (Stages 1–11) grouped by the factor predictor *taxon*. Additionally, it should be noted that since this second model assessed the likelihood of individuals manifesting a systemic stress period, it was run based on a more restricted sample of the 35 NEA and 37 UPMH individuals manifesting linear defects and preserving at least two concurrently forming tooth crowns.

Both models were fit using weakly informative prior distributions (normal with *mean* = 0 and *s.d.* = 1 for intercept and coefficients, exponential (1) for standard deviations) and their performance evaluated with posterior predictive model checking, which compares model predictions with observed data. We ran 4 Markov-Chain-Monte-Carlo (MCMC) chains for each model and obtained coefficient estimates from a total of 8000 post-warm-up samples.

All model parameters reached reliable conversion indicators^68^: a Monte Carlo standard error smaller than 5% of the posterior s.d., an effective posterior sample size greater than 10% of the total sample size, and an $\widehat{R}$ statistic value smaller than 1.01^69^. Using customized code based on Santon et al.^70^, we graphically display the results as the medians of response values across or within the predictors’ and their 95% credible intervals (CIs) of the posterior distributions of fitted values for the population average obtained from the joint posterior distributions of the model parameters [84]. We further used the package *emmeans*^71^ to compute the pairwise contrast odds ratios and their CIs between Neanderthals and UPMH for the likelihood of experiencing a defect for each tooth type from Model 1 and for the likelihood of experiencing a systemic stress period at dental developmental stages 3 and 6 from Model 2.

Effect size strength increases with increasing deviation of ratios from 1, and the robustness of the result increases with decreasing degree of overlap of the 95% compatibility intervals (CIs) with one.

Finally, using the method of finite differences, we estimated the first derivatives of the non-linear trends of Model 2 to identify periods of marked increase or decrease in systemic stress manifestation for each taxon^72^. Such periods are identified as developmental stages where the credible intervals of the first derivatives do not include zero and are highlighted by arrows in the graphical display (Fig. 3).

### Supplementary Information

Supplementary Tables.

Supplementary Information.

## Data Availability

The authors confirm that all data analyzed for this study are included in this published article. For requesting the R code from this study, the first author of the study, Laura Limmer, should be contacted.
