# Supplementary material for: Differences in childhood stress between Neanderthals and early modern humans as reflected by dental enamel growth disruptions
Source: Sci Rep. 2024 May 23;14:11293. doi: 10.1038/s41598-024-61321-x (PMC11116461; doi:10.1038/s41598-024-61321-x)
Supplement: Supplementary file 1 — Supplementary Tables. [file 41598_2024_61321_MOESM1_ESM.docx]

**Differences in childhood stress between Neanderthals and early modern humans as reflected by dental enamel growth disruptions**

**Laura S. Limmer^1,2^, Matteo Santon^3^, Kate McGrath^4,5^, Katerina Harvati^1,2^, Sireen El Zaatari^1,2^***

^1^Paleoanthropology, Senckenberg Centre for Human Evolution and Palaeoenvironment, Institute of Archaeological Sciences, University of Tübingen, Germany.

^2^DFG Center of Advanced Studies ‘Words, Bones, Genes, Tools: Tracking linguistic, cultural and biological trajectories of the human past’.

^3^Ecology of Vision Group, University of Bristol, United Kingdom

^4^Center for the Advanced Study of Human Paleobiology, The George Washington University, USA

^5^CENIEH, Burgos, Spain

* Corresponding author: [sireen.el-zaatari@uni-tuebingen.de](mailto:sireen.el-zaatari@uni-tuebingen.de)

# Supplementary Materials

## Supplementary Table S1.

**Coefficient estimates of Model 1, which investigates the likelihood for single teeth to show a defect in Upper Paleolithic modern humans (UPMH) and Neanderthals (NEA) (Figure 2).** Estimates are based on a Bernoulli distribution with logit-link (see Methods for further details). When the credible intervals do not overlap with zero, the estimate has a higher probability of being non-zero. R^2^ = 0.38, N_NEA_ = 74, N_UPMH_ = 102.

| **Coefficient** | **Mean** | **M. error** | **95 % CI** | |
| --- | --- | --- | --- | --- |
|  |  |  | **Low** | **High** |
| **Population-level effects** | | | | |
| Intercept (UPMH, dd) | -1.29 | 0.35 | -1.97 | -0.60 |
| Tooth type (UPMH, I1) | 1.60 | 0.43 | 0.76 | 2.45 |
| Tooth type (UPMH, I2) | 2.07 | 0.43 | 1.23 | 2.92 |
| Tooth type (UPMH, M1) | 1.11 | 0.35 | 0.41 | 1.81 |
| Tooth type (UPMH, C) | 1.46 | 0.46 | 0.55 | 2.35 |
| Tooth type (UPMH, P3) | 0.38 | 0.46 | -0.53 | 1.29 |
| Tooth type (UPMH, P4) | 0.44 | 0.48 | -0.52 | 1.37 |
| Tooth type (UPMH, M2) | 0.63 | 0.38 | -0.12 | 1.37 |
| Tooth type (UPMH, M3) | -0.45 | 0.48 | -1.41 | 0.48 |
| Hominin (NEA, dd) | 0.20 | 0.43 | -0.65 | 1.05 |
| Tooth type (NEA, I1) | -1.36 | 0.62 | -2.57 | -0.16 |
| Tooth type (NEA, I2) | -0.57 | 0.57 | -1.69 | 0.54 |
| Tooth type (NEA, M1) | -0.69 | 0.49 | -1.65 | 0.27 |
| Tooth type (NEA, C) | 0.40 | 0.56 | -0.71 | 1.51 |
| Tooth type (NEA, P3) | 1.30 | 0.57 | 0.21 | 2.42 |
| Tooth type (NEA, P4) | 2.02 | 0.58 | 0.88 | 3.17 |
| Tooth type (NEA, M2) | 0.87 | 0.49 | -0.08 | 1.84 |
| Tooth type (NEA, M3) | 0.58 | 0.57 | -0.54 | 1.69 |
| **Group-level effects** | | | | |
| Sd (Intercept, Individual ID) | 1.26 | 0.21 | 0.88 | 1.70 |
| Sd (Intercept, Site ID) | 0.76 | 0.25 | 0.23 | 1.25 |

## Supplementary Table S2.

**Pairwise contrasts of defect presence likelihood by tooth type between the hominin groups Upper Paleolithic modern humans (UPMH) and Neanderthals (NEA).**

Pairwise contrasts expressed as odds ratio between specified groups. Effect size strength increases with increasing deviation of ratios from 1, and the robustness of the result increases with decreasing degree of overlap of the 95% compatibility intervals (CIs) with one (see Methods for more details).

| **Contrasts** | **Tooth type** | **Odds ratio** | **95 % CI** | |
| --- | --- | --- | --- | --- |
|  |  |  | **Low** | **High** |
| UPMH/NEA | Average tooth | 0.62 | 0.23 | 1.20 |
|  | dd | 0.82 | 0.28 | 1.76 |
|  | I1 | 3.22 | 0.48 | 9.72 |
|  | I2 | 1.46 | 0.21 | 4.20 |
|  | M1 | 1.64 | 0.39 | 3.90 |
|  | C | 0.56 | 0.08 | 1.61 |
|  | P3 | 0.23 | 0.03 | 0.62 |
|  | P4 | 0.11 | 0.02 | 0.32 |
|  | M2 | 0.35 | 0.08 | 0.82 |
|  | M3 | 0.46 | 0.07 | 1.28 |

## Supplementary Table S3.

**Pairwise contrasts of defect presence likelihood by tooth type within the hominin groups Upper Paleolithic modern humans (UPMH) and Neanderthals (NEA).**

Pairwise contrasts expressed as odds ratio between specified groups. Effect size strength increases with increasing deviation of ratios from 1, and the robustness of the result increases with decreasing degree of overlap of the 95% compatibility intervals (CIs) with one (see Methods for more details).

| **Contrasts** | | **Odds ratio** | **95 % CI** | |
| --- | --- | --- | --- | --- |
| **Hominin group** | **Tooth types** |  | **Low** | **High** |
| UPMH | dd-I1 | 0.2031 | 0.0688 | 0.419 |
|  | dd-I2 | 0.1262 | 0.0402 | 0.260 |
|  | dd-M1 | 0.3328 | 0.1388 | 0.610 |
|  | dd-C | 0.2324 | 0.0709 | 0.506 |
|  | dd-P3 | 0.6807 | 0.2011 | 1.499 |
|  | dd-P4 | 0.6373 | 0.1855 | 1.450 |
|  | dd-M2 | 0.5335 | 0.2095 | 1.026 |
|  | dd-M3 | 1.5543 | 0.4156 | 3.505 |
|  | I1-I2 | 0.6271 | 0.1462 | 1.484 |
|  | I1-M1 | 1.6331 | 0.4750 | 3.655 |
|  | I1-C | 1.1441 | 0.2573 | 2.923 |
|  | I1-P3 | 3.3573 | 0.7516 | 8.618 |
|  | I1-P4 | 3.1826 | 0.6271 | 8.229 |
|  | I1-M2 | 2.6401 | 0.7155 | 6.060 |
|  | I1-M3 | 7.6486 | 1.5953 | 20.789 |
|  | I2-M1 | 2.6242 | 0.7443 | 5.899 |
|  | I2-C | 1. 8426 | 0.4439 | 4.666 |
|  | I2-P3 | 5.3490 | 1.2282 | 14.175 |
|  | I2-P4 | 5.0673 | 1.0976 | 13.106 |
|  | I2-M2 | 4.2358 | 1.1268 | 9.657 |
|  | I2-M3 | 12.2575 | 2.4138 | 33.328 |
|  | M1-C | 0.7024 | 0.1668 | 1.640 |
|  | M1-P3 | 2.0524 | 0.5395 | 4.813 |
|  | M1-P4 | 1.9356 | 0.4861 | 4.620 |
|  | M1-M2 | 1.6175 | 0.5665 | 3.169 |
|  | M1-M3 | 4.6913 | 1.0865 | 11.380 |
|  | C-P3 | 2.9388 | 0.5456 | 7.817 |
|  | C-P4 | 2.7624 | 0.5795 | 7.545 |
|  | C-M2 | 2.916 | 0.5644 | 5.472 |
|  | C-M3 | 6.6832 | 1.2479 | 18.961 |
|  | P3-P4 | 0.94607 | 0.1738 | 2.517 |
|  | P3-M2 | 0.78886 | 0.2179 | 1.871 |
|  | P3-M3 | 2.2889 | 0.4490 | 6.311 |
|  | P4-M2 | 0.8393 | 0.2039 | 2.022 |
|  | P4-M3 | 2.4399 | 0.4397 | 6.825 |
|  | M2-M3 | 2.9017 | 0.7752 | 7.173 |
| NEA | dd-I1 | 0.7829 | 0.1630 | 2.096 |
|  | dd-I2 | 0.2236 | 0.0536 | 0.531 |
|  | dd-M1 | 0.6594 | 0.2133 | 1.403 |
|  | dd-C | 0.1570 | 0.0421 | 0.350 |
|  | dd-P3 | 0.1863 | 0.0553 | 0.427 |
|  | dd-P4 | 0.0858 | 0.0224 | 0.196 |
|  | dd-M2 | 0.2232 | 0.0709 | 0.472 |
|  | dd-M3 | 0.8740 | 0.2296 | 1.956 |
|  | I1-I2 | 0.2845 | 0.0426 | 0.840 |
|  | I1-M1 | 0.8555 | 0.1512 | 2.300 |
|  | I1-C | 0.2019 | 0.0313 | 0.568 |
|  | I1-P3 | 0.2394 | 0.0390 | 0.673 |
|  | I1-P4 | 0.1089 | 0.0168 | 0.308 |
|  | I1-M2 | 0.2858 | 0.0443 | 0.772 |
|  | I1-M3 | 1.1220 | 0.1633 | 3.104 |
|  | I2-M1 | 2.9505 | 0.7535 | 7.150 |
|  | I2-C | 0.7021 | 0.1404 | 1.794 |
|  | I2-P3 | 0.8409 | 0.1685 | 2.157 |
|  | I2-P4 | 0.3821 | 0.0818 | 0.978 |
|  | I2-M2 | 1.0017 | 0.2321 | 2.390 |
|  | I2-M3 | 3.9227 | 0.8773 | 9.970 |
|  | M1 - C | 0.2368 | 0.0596 | 0.539 |
|  | M1-P3 | 0.2825 | 0.0803 | 0.642 |
|  | M1-P4 | 0.1293 | 0.0354 | 0.297 |
|  | M1-M2 | 0.3374 | 0.1117 | 0.716 |
|  | M1-M3 | 1. 3218 | 0.4162 | 2.935 |
|  | C-P3 | 1.1884 | 0.2622 | 2.856 |
|  | C-P4 | 0.5468 | 0.1309 | 1.312 |
|  | C-M2 | 1.4208 | 0.3960 | 3.246 |
|  | C-M3 | 5.5698 | 1.5777 | 13.701 |
|  | P3-P4 | 0.4555 | 0.1123 | 1.098 |
|  | P3-M2 | 1.1960 | 0.3402 | 2.691 |
|  | P3-M3 | 4.6736 | 1.3221 | 11.330 |
|  | P4-M2 | 2.6137 | 0.7349 | 6.002 |
|  | P4-M3 | 10.2942 | 2.4182 | 24.786 |
|  | M2-M3 | 3.9152 | 1.1480 | 8.577 |

## Supplementary Table S4.

**Coefficient estimates of model 2, which investigates the likelihood of systemic stress occurrences in UPMH and NEA through developmental stages (Figure 3).**

Estimates are based on a Bernoulli distribution with logit-link (see Methods for further details). When the credible intervals do not overlap with zero, the estimate has a higher probability of being non-zero. R^2^ = 0.38, N_NEA_ = 35, N_UPMH_ = 37.

| **Coefficient** | **Mean** | **M. error** | | **95 % CI** | |
| --- | --- | --- | --- | --- | --- |
|  |  |  |  | **Low** | **High** |
| **Population-level effects** | | | | | |
| Intercept (UPMH) | -3.24 | 0.49 | -4.24 | | -2-29 |
| Hominin (NEA) | 0.59 | 0.59 | -0.56 | | 1.73 |
| Unpenalised weight (UPMH) | 0.05 | 0.97 | -1.87 | | 1.95 |
| Unpenalised weight (NEA) | 0.12 | 0.99 | -1.82 | | 2.09 |
| **Smooth terms** | | | | | |
| Sd of smooth weights (UPMH) | 5.04 | 2.61 | 1.48 | | 11.45 |
| Sd of smooth weights (NEA) | 8.75 | 3.74 | 3.40 | | 17.82 |
| **Group-level effects** | | | | | |
| Sd (Intercept, Individual ID) | 0.41 | 0.33 | 0.01 | | 1.20 |
| Sd (Intercept, Site ID) | 1.45 | 0.37 | 0.72 | | 2.21 |

## Supplementary Table S5.

**Pairwise contrasts of stress event likelihood by developmental stage between the hominin groups Upper Paleolithic modern humans (UPMH) and Neanderthals (NEA).**

Pairwise contrasts expressed as odds ratio between specified groups. Effect size strength increases with increasing deviation of ratios from 1, and the robustness of the result increases with decreasing degree of overlap of the 95% compatibility intervals (CIs) with one (see Methods for more details).

| **Contrasts** | **Developmental Stage** | **Odds ratio** | **95 % CI** | |
| --- | --- | --- | --- | --- |
|  |  |  | **Low** | **High** |
| UPMH/NEA | 3 | 4.7968 | 0.3492 | 18.554 |
|  | 6 | 0.075 | 0.00781 | 0.257 |
